# A New Framework for Narcissism in Health Psychology and Psycho-Oncology

**DOI:** 10.3389/fpsyg.2020.01182

**Published:** 2020-06-03

**Authors:** Gaia Perego, Valentina E. Di Mattei

**Affiliations:** ^1^Department of Psychology, University of Milano-Bicocca, Milan, Italy; ^2^School of Psychology, Vita-Salute San Raffaele University, Milan, Italy; ^3^Division of Neuroscience, Clinical and Health Psychology Unit, IRCCS San Raffaele Scientific Institute, Milan, Italy

**Keywords:** cancer, humanization of medicine, body image, self-esteem, narcissism

## Introduction

The extant literature highlights the impact of Narcissistic Personality Disorder (NPD) on health conditions, treatment adherence and interactions with healthcare workers (Quirk et al., 2016; Di Mattei et al., 2018). Specifically, people with NPD show an increased risk for organic disease (Edelstein et al., 2012; Quirk et al., 2016), low treatment compliance (Pontiroli et al., 2007) and a higher use of medical services (Powers et al., 2014). In addition, NPD is associated with harmful health behaviors, such as alcohol and drug use (Luhtanen and Crocker, 2005; MacLaren and Best, 2013; Hill, 2016), risky sexual behavior (Lavan and Johnson, 2002; Martin et al., 2013), and dangerous driving (Hill, 2016). Finally, NPD has been frequently found among people resorting to cosmetic and plastic surgery (Shridharani et al., 2010; Belli et al., 2013; Agostino et al., 2018).

Nevertheless, considering the high rates found in the general population, NPD has been frequently neglected by psycho-oncology.

Research on NPD is in fact limited by the lack of an exhaustive and univocal conceptualization of the construct (Cain et al., 2008), which is mainly due to differences between phenotypic and taxonomic models and the heterogeneity of the definitions used (Di Pierro and Madeddu, 2018). In fact, the Diagnostic and Statistical Manual of Mental Disorders (DSM) underlined the grandiose and arrogant behavioral manifestations of narcissism, overlooking its vulnerable characteristics, which are most frequently found in clinical practice. The reductionism of the DSM diagnosis led to the underestimation of the prevalence rates of the disorder itself. In addition, the vulnerable manifestations of narcissism received little attention from researchers (Di Pierro and Madeddu, 2020). Moreover, Pincus and Lukowitsky (2010) defined narcissism as the ability of the individual to maintain a relatively positive self-image through self-regulation and interpersonal regulation processes. Narcissism, therefore, can be regarded as a trait present in each individual.

Recently, the definition of DSM-based personality disorder has been revised, questioning its categorical classification and other shortcomings. In particular, NPD has been analyzed focusing on more peculiar themes including vulnerability, grandiosity and self-esteem issues. Indeed, when not rigid and pervasive, narcissism allows the individual to effectively deal with any frustrations by maintaining adequate self-esteem levels and regulating negative emotions (Di Pierro and Madeddu, 2020). These changes enable a new interpretation of several aspects.

## Self-Esteem and Cancer

Severe medical disease can exacerbate fears of neglect and abandonment, as well as feelings of shame and guilt, leading to behavioral regression (Meyer and Block, 2011) and to the manifestation of narcissistic vulnerabilities. In fact, finding out to be ill often represents a moment of crisis with respect to the usual functioning. This is particularly true when referring to a life-threatening diagnosis, such as cancer, which deeply affects the sense of self. Specifically, it affects multiple sources of self-esteem regulation, such as physical appearance, autonomy, independence, power, and social roles.

A peculiar cause of self-esteem impairment is related to alterations in perceived body image (Harter, 1999), as attractiveness is associated with inter-personal success and it is an important determinant of social prestige and acceptance (Marium and Iftikhar, 2014). Body image issues can be detrimental especially for women, as men are more likely to focus their body awareness on functional capabilities, whereas women are more prone to put emphasis on appearance (Halliwell and Dittmar, 2003). Attacks to feminine identity can alter self-esteem, especially in cultures that value traditional gender roles. Compared to men, women seem in fact to be more distressed by cancer-related aesthetic symptoms (Nozawa et al., 2013).

In this regard, a crucial aspect involves the profound impact of oncological treatment. Remarkably, understanding when and how to think psychologically about the patient's situation is beneficial for oncologists, because neglecting mental health compromises patient care (McFarland and Hlubocky, 2019).

For instance, chemotherapy is often associated with cosmetic side effects, including alopecia, paleness, dry skin, and weight loss or gain (Amiel et al., 2009). These adverse reactions can represent for patients a source of distress comparable to the disease itself (Di Mattei et al., 2016, 2017). Notably, the aesthetic consequences of therapies, such as hair loss, can be experienced in terms of a real trauma, especially by the female population (Lemieux et al., 2008), leading to anxiety and depression symptoms (Stafford et al., 2015). In turn, such detrimental effects provoke a significant impairment of self-esteem and perceived body image (Pinar et al., 2012; Di Mattei et al., 2017). In fact, illness often becomes visible with the beginning of treatment, which is experienced as a period of body deterioration (Quintard and Lakdja, 2008) and loss of social and emotional identity (Amiel et al., 2009). Analogously, surgery can deeply affect body image and self-esteem (Callahan, 2005; Melissant et al., 2019), which are negatively associated with a decline in quality of life (Manos et al., 2005). Surgery can damage body integrity with scars or even permanently alter highly visible body parts (e.g., head and neck surgery), functional organs (e.g., colorectal surgery) and symbols of femininity (e.g., breast surgery).

Indeed, the main adverse sequelae spontaneously reported by female cancer patients deal with physical changes, which are described as “painful narcissistic effects” (Amiel et al., 2009). Hair loss and mastectomy are frequently defined as a mutilation (Amiel et al., 2009; Zannini et al., 2012), and they are associated to difficulties in recognizing oneself in the mirror. In this regard, wearing a wig helps women to maintain a sense of identity (Zannini et al., 2012).

Another important aspect concerns social interactions. As hair loss can expose patients to other people judgment, sometimes wearing a wig is a forced choice to hide the disease. In this regard, women report that they wear wigs mainly outside domestic walls because they are embarrassed and they want to avoid pity and compassion from other people (Zannini et al., 2012). Women also declare that people start to look at them differently when they show treatment side effects (Amiel et al., 2009) and they are worried about being deemed unattractive by others (Nozawa et al., 2013).

## Aesthetic Care Interventions

In this perspective, the introduction of aesthetic care interventions inside hospitals enables a global vision of the patient, not just by “curing” the disease, but also by “taking care” of the person as a whole. These programs can provide female cancer patients with an opportunity to regain a positive contact with their bodies, which have been altered by aggressive therapies, thus reconnecting with their beauty and femininity (Di Mattei et al., 2017). The focus of these interventions is in fact the recovery of adequate self-esteem levels, normally present before cancer diagnosis.

A growing number of studies investigated the efficacy of these programs on women's well-being, both by means of qualitative (Amiel et al., 2009; Zannini et al., 2012) and quantitative data (e.g., Titeca et al., 2007; Quintard and Lakdja, 2008; Park et al., 2015; Richard et al., 2019). The literature indicates that participating in aesthetic care programs could determine an improvement in body image, anxiety, self-esteem, and the quality of social interactions (Quintard and Lakdja, 2008; Richard et al., 2019), thus facilitating a better adjustment to the disease.

## Health in the Mirror

Within this framework, a psychosocial support program named “Health in the Mirror” was developed in Northern Italy. It is an intervention for female cancer patients aimed at helping to manage appearance-related side effects resulting from cancer and its treatment. The program includes an initial psychological assessment and three group sessions that take place on a weekly basis. After an initial make-up and wig tutorial, each patient receives a personalized make-up session and is given the possibility to choose a wig to keep. A photographer captures patients' portraits before and after their personalized treatment. A dermatologist gives a lecture on how to treat the skin and body during cancer treatments. Afterwards, a nutritionist illustrates the guidelines for a healthy diet, suggesting how to counter some of the potential side effects of cancer treatment. Moreover, a fashion stylist offers a customized consultation on the use of colors to match each patient's skin tone. During the last session, a team of psychologists leads a group discussion focusing on patients' cancer experience and their experience of taking part in the program.

Noticeably, a quantitative assessment showed that participating in the Health in the Mirror program fostered a significant reduction in depressive symptoms, anxiety and body image issues, both immediately at the conclusion of the program and 3 months later. Remarkably, self-esteem levels showed an improvement at the 3-months follow-up (Di Mattei et al., 2017).

## Discussion

Interventions aimed at rebuilding body image following its deterioration seem to favor a normal regulation of self-esteem. This allows the restoration of adequate levels of narcissism. Indeed, being proud and satisfied with oneself and one's own image is not a sign of narcissistic pathology, but rather a form of self-acceptance required for normal development (Thomaes et al., 2010; Lipowska and Lipowski, 2015). For instance, wearing a wig is considered crucial to identity restoration following cancer treatment (Zannini et al., 2012). In addition, photo shooting allows participants to visualize the bodily appearance effects of the beauty care intervention for a second time and to share professionally edited photos, thereby likely fostering increases in self-esteem (Richard et al., 2019).

In general, maintaining a high level of self-esteem predicts better psychological adjustment to cancer and its treatment, in terms of a higher quality of life (Bartoces et al., 2009) and reduced levels of distress, anxiety and depression (Kobayashi et al., 2008; da Mata et al., 2016; Yang et al., 2019). Therefore, in addition to the best possible medical therapy, the improvement of patients' quality of life must be a standard of care in oncology, as it promotes compliance to treatment (Cheville et al., 2015). In this perspective, psychosocial interventions should be combined with standard oncological care (Di Mattei et al., 2018).

In conclusion, as far as narcissistic themes are concerned, it is necessary to go beyond the underestimation often found in oncology and the medical environment. Although many authors agree on the existence of healthy and pathological narcissistic traits, the nature of this distinction is not yet clear. Finally, despite the propensity of psychology to address only profound issues, dealing with apparently trivial themes can enhance patients' self-esteem.

## Author Contributions

GP wrote the first draft of the manuscript. VD wrote sections of the manuscript. Both authors contributed to the manuscript revision, read, and approved the submitted version.

## Conflict of Interest

The authors declare that the research was conducted in the absence of any commercial or financial relationships that could be construed as a potential conflict of interest.
